# Evaluation of a new set of indicators for mental health care implemented in Madhya Pradesh, India: a mixed methods study

**DOI:** 10.1186/s13033-020-0341-4

**Published:** 2020-02-10

**Authors:** Shalini Ahuja, Azaz Khan, Lucy Goulding, Rachel Kaur Bansal, Rahul Shidhaye, Graham Thornicroft, Mark Jordans

**Affiliations:** 1 Centre for Global Mental Health, Health Services and Population Research Department, Institute of Psychiatry, Psychology & Neuroscience, King’s College London, London, UK; 2 Programme for Improving Mental Health Care, Sangath, Bhopal India; 3 Centre for Implementation Science, Institute of Psychiatry, Psychology & Neuroscience, King’s College London, De Crespigny Park, London, SE5 8AF UK; 4 City, University of London, London, UK; 5 Public Health Foundation of India, New Delhi, India

**Keywords:** Evaluation, Mental health information systems, Global mental health, Mixed methods research, Mental health indicators, India

## Abstract

**Background:**

Mental health information systems are, in general, inadequate and unreliable in India. We have developed key mental health indicators for measuring mental health service coverage in primary care. This study aims to evaluate the use of these new indicators in seven health care facilities in Sehore District of Madhya Pradesh in India.

**Methods:**

The study employed a mixed methods approach. We conducted: a qualitative study (n = 26) with health workers, Health Management Information Systems (HMIS) staff, project coordinators and supervisors; a review of case records (n = 61 at time 1 and n = 74 at time 2); and a structured questionnaire (n = 26) with health workers. The performance, user-friendliness, appropriateness, perceived utility and sustainability of the use of new mental health indicators was assessed.

**Results:**

High levels of completion, and correctness of completion, of the new mental health indicators were found for the case records. The simplicity of the forms, as well as technical support from the project team, contributed to acceptability and feasibility of implementation. Perceived sustainability of the new forms was, however, affected by the overstretched primary care staff. Further work is needed to support the integration of mental health with routine HMIS.

**Conclusion:**

This study demonstrated that the implementation of key mental health service delivery indicators in Sehore District primary care facilities was feasible. Technical assistance was imperative in maintaining the performance of the indicators over the two studied time points. The integration of mental health indicators in routine health information systems, and political buy-in, are needed to monitor and sustain community mental health programmes in India.

## Background

India’s new Mental Health Care Act 2017 [1] and the Mental Health Policy 2014 are remarkable initiatives to address the needs of people living with mental health conditions. Despite recent developments, unmet care and treatment needs continue to be a concern in India [2]. Scaling-up of mental health care, for example to increase the availability of evidence-based mental health interventions at the community level, is advocated to reduce the huge gap amongst those who need and those who receive effective treatment for mental illnesses [3]. Integration of mental health in primary care is needed and, in order to strengthen mental health services, it is important to have a robust system of collecting, analysing and using routine data [4, 5].

The World Health Organisation is committed to the development and strengthening of information systems for mental health in Low- and Middle-Income Countries (LMICs) [6]. In India, the Ministry of Health and Family Welfare has a robust, web-based health management information system (HMIS) for monitoring national health programmes. This HMIS primarily caters to the needs of maternal and child health services [4]. However, routine data for services related to people with mental disorders have a weak presence in the national HMIS in India, as in other LMICs [4, 7]. In general, routine information on mental health service delivery is not collected because there are no indicators included in the routine information systems, and when they are, the information collected is often of poor quality. Therefore, there is a need to develop and utilise mental health indicators which are feasible and acceptable to health care professionals over time [5, 8].

In this context, the aim of this paper was to evaluate the use of the newly introduced mental health indicators in the context of integrated primary mental health care services in the Indian state of Madhya Pradesh. Specifically, we evaluated the performance, user-friendliness, appropriateness, and the perceived utility and sustainability of a new set of indicators for routine monitoring of mental health care within primary care facilities in Sehore District of Madhya Pradesh.

## Methods

### Study setting

The Department of Health (DoH) of Madhya Pradesh is responsible for the overall management and development of mental health plans and their service delivery. It provides mental health services through: (a) two mental hospitals in Indore and Gwalior; (b) general hospitals providing secondary and tertiary mental health care; and (c) the District Mental Health Programme (DMHP), which focuses on delivering mental health at primary care and also provides outreach services in the communities. The DMHP is functional only in two districts, i.e. Sehore and Chhindwara. However, the plans are to scale the DMHP to five more districts and then to the entire 51 districts of Madhya Pradesh.

Health facilities delivering primary care in the Sehore District were selected for developing and implementing the new mental health indicators. The Sehore District was selected due to its poor general health indicators, particularly with regards to child and reproductive health rankings [9]. A functional district mental health programme was another key reason to choose Sehore District as the study site.

Through the Programme for Improving Mental Health Care (PRIME), a mental health service delivery platform was created at these facilities, within which these indicators were developed and sequentially tested. The PRIME programme within Sehore District implemented and improved service delivery processes related to awareness, detection, treatment and recovery of people with Depression, Psychosis and Alcohol Use Disorders, and enabled the processes of supervision, and development of the new HMIS [10].

#### Emerald project

As a part of the Emerging Mental Health Systems for Low- and Middle-Income Countries (Emerald) project, which aimed at strengthening mental health systems in LMICs, we developed context-specific mental health indicators measuring mental health care needs, utilisation, quality and financial protection for primary care health facilities in Sehore district of Madhya Pradesh [11]. Mental health care needs, utilisation and quality of services are key contributors to the concept of effective coverage, which is the proportion of people with mental disorders receiving quality care.

#### Context

The DMHP envisages the provision of community mental health services by facilitating integration of mental health care by decentralising the treatment from specialised mental hospitals thereby promoting mental health care for all.

There is ample policy and legislative context through India’s first mental health policy (2014), the Mental Health Care Bill 2017 and the National Mental Health Programme for the design and implementation of the DMHP.

The elements of DMHP include the provision of clinical services, the training of general health care providers, information and communication programmes and the provision of community data and experience to prepare for future planning.

A psychiatrist, a clinical psychologist, a psychiatric social worker, a community nurse/worker, a programme manager, a case management coordinator and a record keeper are required to be a part of the DMHP team at a district level [12]. Out of the 640 districts, by 2014, DMHP was implemented in 127 districts [13]. Within Madhya Pradesh, DMHP was implemented between 2003 and 2004 in the Sehore district [12]. The current evaluations of DMHP suggest its effective implementation [13, 14]. The DMHP is often unable to hire the entire district team and depends primarily on primary health care physicians and community nurses who are unable to fulfil the programme’s vision with their growing workload and lack of motivation [15]. In some districts (such as in Sehore) as a part of DMHP, outreach camps are provided by the psychiatrist and the psychologist to promote mental health awareness and reduce stigma [16]. Overall, shortage and poor distribution of mental health professionals in DMHP is a major challenge in achieving the goal of reducing the treatment gap of mental disorders. As the DMHP is undergoing expansion, it is proposed to develop a central coordinating and monitoring cell with periodic site visits to ensure appropriate supervision, mainly because the evidence on the supervision practices is still patchy [17]. Therefore, further research is needed to understand the barriers and facilitators of implementing mental health services, especially at primary levels in India [18].

Routine information systems in states like Madhya Pradesh contain indicators on major and minor mental disorders, as part of monitoring for the DMHP. However, this system of classifying mental disorders into major (relates to severe mental disorders like schizophrenia) and minor (relates to common mental disorders such as depression) is outdated and needed revision [16]. Most of the data on these indicators was collected from district hospitals, and barring some data on outreach services, no mental health data was available from primary health centres and community health centres.

The new forms collecting data on the mental health indicators were implemented in the PRIME scale up facilities, namely Budhni, Ichhawar, Nasrullaganj, Rehti, Ladhkui, and Ashta civil hospital and Sehore district hospital. There was no notable difference in the facilities in terms of infrastructure or human resources. Except for Sehore district hospital, where a part time psychiatrist visited once a week and there was a regular psychologist, in all other health facilities nurses and medical doctors were key focal points for diagnosis and management of three priority mental disorders, i.e. Depression, Psychosis and Alcohol Use Disorders.

Due to the motivation of some of the local leaders, establishing mental health service delivery within some of the facilities was particularly easy. However, other primary care facilities were reluctant in integrating mental health programmes within their primary care service delivery system.

Regular meetings of the PRIME co-ordination team with the facility in charge and other medical officers explored measures to mitigate these challenges, which resulted in the refresher on the job training of all the nurses in these facilities. In health facilities such as Rehti and Ichhawar, mental health service delivery was incepted well, whereas in Ladhkui it was affected by administrative issues. In Nasrullaganj community health facility, the service delivery inception was received well by the staff but suffered due to frequent leadership changes and weak inter-team co-ordination. In Ashta, the Block Medical Officer (facility in charge) provided a full-time nurse to manage the service delivery and reporting of the mental health programme. In facilities such as Budhni, PRIME struggled to deliver regular mental health services despite regular meetings with the nurses and the medical officer.

### Indicator development and implementation

New mental health indicators were developed in a phased manner across six countries using: (a) a situational analysis tool to assess the status of the mental health information system in India; (b) a prioritisation exercise to rank key mental health indicators for the Sehore District; and (c) consultative workshops to review if the selected indicators were ready to be implemented [11]. The final set of health facility-level mental health indicators developed for Sehore District included indicators measuring: (a) diagnosis made by the primary care physicians (of depression, alcohol use disorders and psychosis); (b) exact diagnosis confirmed by the psychiatrist; (c) severity of disorder; (d) treatment administered (including both psychosocial and pharmacological interventions); (e) referrals to tertiary care facilities; (f) re-admissions; and (g), follow-up. Subsequently, these indicators were developed into forms for data collection.

Within the PRIME India, packages of care for mental health at primary care were delivered through district mental health care plans [18]. In the scale up phase of PRIME, the new forms collecting data on Depression, Psychosis and Alcohol Use Disorders were administered by nurses at five community health centres (Budhni, Ichhawar, Nasrullaganj, Rehti, and Ladhkui), one civil hospital (Ashta) and one district hospital (Sehore). Table 1 provides more information on each of these facilities. The data on the new indicators using these forms were collected for about a year. For the purpose of this study, use of these indicators was assessed at two time points.

**Table 1:** Indicator implementation facilities

Synod	Health Facility (PRIME scale up facilities)	Catchment area (in km)	Total number of patients with psychosis, depression and alcohol use disorders registered (July 2016 to Sept 2017)
1	Ichhawar CHC	50	38
2	Budhni CHC	20	33
3	Nasrullaganj CHC	55	123
4	Ashta Civil Hospital	42	122
5	Rehti CHC	22	37
6	Ladhkui CHC	40	80
7	Sehore District Hospital	80	380

*CHC* community health centre

The implementation of these new forms collecting data on the health facility indicators included development of registers for record keeping, and a 3-day training of nurses, medical officers and frontline health workers on mental health information systems and other mental health programme activities. Implementation of the mental health programme also involved monthly supervision by PRIME programme coordinators [19]. To ensure sustainability, support by the programme coordinators was gradually weaned off by the end of the first year of implementation, after which the nurses mainly managed the mental health programme at the facilities.

### Study design

A sequential, exploratory mixed methods approach was used for this study. The results are explored qualitatively and then interpreted through quantitative follow-up research [20]. Qualitative data were collected using semi-structured interviews from April 2017 to September 2017.

Quantitative data were collected by reviewing patient records at two times points (T1 in October 2016 and T2 in August 2017) with a 9-month interval, as well as using a face-to-face structured interview with health workers again at T1 and T2.

### Sampling

In the qualitative study, 26 participants including nurses/records staff (n = 10), HMIS staff (n = 3), medical officers (n = 6), supervisors (n = 3), and programme co-coordinators (n = 4), were recruited based on their roles in delivering or managing mental health services.

For the quantitative component, patient records for the month of October 2016 and August 2017 were accessed and reviewed. All the patient records were selected at both these time points, so no sampling was needed. At time 1 (T1), 61 patient records, and at time 2 (T2) another 74 patient records, were included in the study.

For the structured questionnaire, at T1, 16 respondents (n = 16, including 9 nurses and 7 supervisors) delivering mental health services at these facilities were included. At T1, one of the nurse/records staff (out of a total of 10) was not available for participation in the questionnaire. The same health workers were again involved at T2. At T2, a total of 9 respondents were included, comprising of 7 out of 9 nurses and 2 supervisors, who agreed to participate in the questionnaire.

### Procedures

Regarding the qualitative component, a semi-structured topic guide was developed based on implementation outcomes, mainly performance, appropriateness, user-friendliness, perceived utility (acceptability, feasibility), and sustainability of implementing new forms and indicators. These implementation outcomes are adapted from Proctor and colleagues and are described in Table 2 [21]. Topic guides were developed both in English and Hindi by one of the authors (SA) and a local researcher. To begin with, the topic guides assessed generic feasibility of these indicators, followed by other implementation outcomes assessing the actual use of these indicators. Some other implementation outcomes such as penetration were not assessed at this stage, as these outcomes are better suited to be assessed at later stages of implementation. The interviews with health workers were conducted in health facilities in Sehore and with programme co-ordinators and supervisors in the PRIME offices in Sehore. These were individual in-depth interviews, and each lasted for about one hour. Interviews were recorded and later transcribed. Back translations were carried out for interviews conducted in Hindi.

**Table 2:** Assessment of implementation outcomes for the new mental health information system. Adapted from Proctor, 2010

Implementation outcome	Level of analysis	Implementation stage	Method and measurement
Performance (completeness, correction of completeness)	Service users	At two time points (9-month interval)	Service user’s records
User- friendliness and appropriateness	Health workers, medical officers Health Managers/Programme coordinator/Supervisors	Qualitative: mid and towards the end of year one of implementation Quantitative: at two time points (9-month interval)	Qualitative interviews Structured questionnaires
Acceptability	Health workers, medical officers Health Managers/Programme coordinator/Supervisors	Qualitative: mid and towards the end of year one of implementation Quantitative: at two time points (9-month interval)	Qualitative interviews Structured questionnaires
Feasibility	Health workers, medical officers Health Managers/Programme coordinator/Supervisors	Qualitative: mid and towards the end of year one of implementation Quantitative: at two time points (9-month interval)	Qualitative interviews Structured questionnaire
Sustainability	Health workers, medical officers Health Managers/Programme coordinator/Supervisors	Qualitative: mid and towards the end of year one of implementation Quantitative: at two time points (9-month interval)	Qualitative interviews Structured questionnaire

For the quantitative component, a structured questionnaire was developed. The questionnaire encompassed several implementation outcomes and was designed to capture the perceptions of utility, appropriateness, and user friendliness [21]. First, as part of a larger programme of work in the Emerald project, a cross-country structured questionnaire was developed which was later contextualised for India and translated to Hindi. For reviewing the patient records, performance was calculated by assessing the completion rates and the correctness of completion rates of the patients’ records. Completion was scored, by the first author and a country researcher, as either partially complete or fully complete. Similarly, the responses for correctness of completion rate were further divided into illogical, illegible and incomplete responses, and scored by the same two researchers.

### Data analysis

Qualitative data analysis followed thematic analysis principles [22]. Initial open codes were descriptive and were grouped into broadly conceptually coherent categories using NVIVO-10 software. These were considered ‘parent themes’ guiding further coding. Open codes were then deductively mapped out against the existing framework called Performance of Routine Information System Management (PRISM) [23] to develop themes. Hence this existing framework was used for deductive development of themes. Finally, the data were populated in an Excel spreadsheet, with themes from the categories described in the PRISM framework, namely technical, organisational, and behavioural determinants. Technical determinants describe the data collections forms, processes and systems; organisational determinants cover the resources, roles and responsibilities; and behavioural determinants explain the knowledge, skills, motivation and attitudes of health workers who collect and use data.

Quantitative data were analysed using descriptive statistics using percentage of responses.

### Ethics review

Ethical permission from the review boards of Public Health Foundation of India (TRC-IEC-200/13; TRC-IEC-201/13; TRC-IEC-202-13) and King’s College London (PNM-1314-4) was granted for the study. Informed written consent was provided by all participants. Anonymised case records were used for the purpose of analysis.

## Results

Using the PRISM framework, we report technical, organisation and behavioural components affecting the implementation of the new mental health indicators in Sehore District. Results from the case records review and the structured questionnaire, are embedded in the qualitative themes. Some of the overall findings from the case records review and the structured questionnaire are presented in a cross-country paper [24]. However, this results section delves into the specification of various sub items of the questionnaire and patients’ records. As stated above, these findings are nested into the relevant qualitative themes, to enhance the contextual understanding of the use of these new mental health indicators in the health facilities in Sehore.

### Technical determinants

#### Completion of data in the health facilities

All the health workers interviewed considered that the new forms with mental health data were easy to understand and simple to complete. Through the structured questionnaires, the items assessing willingness to complete new forms, easiness of the task/user friendliness, and the relevance/appropriateness of using new mental health forms, remained higher than 85% across both time points when the response was selected as either agreed or strongly agreed (see Table 4).

Across the two times points, after reviewing 61 and 74 patient records, respectively, we calculated the completion rates for all health facility indicators (see Table 3). Overall, completion rates for mental health service indicators were found to be either slightly reduced or they remained constant across the two time points. Completion rates of indicators on diagnosis, exact diagnosis (confirmed by a psychiatrist) and treatment administered were above 85%, both at T1 and T2, with slight reduction in the completion rates at T2 as compared to T1. The indicator on severity noted a reduction in completion at T2 as compared to T1. For indicators on referral and follow-up, only the patients who were referred and followed up were recorded by the health workers, therefore these indicators could not be compared with other indicators.

**Table 3:** Completion and correction of completion rates of mental health service indicators

Criteria	Number of patients diagnosed with three priority disorders, n (%)	Number of patients where the diagnoses was confirmed by a psychiatrist, exact diagnosis, n (%)	Severity, n (%)	Treatment administered (either psychological or pharmaceutical treatments), n (%)	Referral, n (%)	Follow up, n (%)	
Completion in the new formats							
T1 N = 61	61 (100)	49 (80.0)	61 (100)	60 (98.0)	2(100)^a^	41 (100.0)	
T2 N = 74	65 (87.8)	63 (85.1)	29 (39.1)	65 (87.8)	4 (100)^a^	3 (100.0)	

Criteria	Time point	Diagnosis	Exact diagnosis	Severity	Treatment administered	Referral	Follow up
Correctness of completion	T1	60 (98.0)	46 (94.0)	60 (98.0)	42 (70.0)	2 (100)^a^	41 (100)
	T2	64 (98.4)	55 (87.3)	29 (100)	64 (98.4)	4 (100)^a^	3 (100)
Data quality issue	T1	1 (partially written)	3 (illogical)	1 (Illegible)	18 (illogical 8, partially complete 9, Illegible 1)	0	0
	T2	1 (partially written)	8 (partially complete)	0	1 (Illegible)	0	0

^a^The response options and the denominators for these items were different from the other items, these are therefore not included in the summarised results

#### Correctness of completed mental health indicators

During the qualitative interviews, health workers reported that their understanding of the forms has improved over time, and as per most of the supervisors and programme coordinator the quality of data reported by the respondents is generally accurate. Primary care practitioners elaborated that the monthly reporting initially took longer to reach the directorate of health services at state level, however the process of transferring reports also streamlined over time.

The accuracy with which the case records were completed is further elaborated through the quantitative findings. Through the structured questionnaire, confidence of health workers in the use of data using the new format was reported to have increased from 43% at T1 to 78% at T2, when the response was selected as either ‘agree’ or ‘strongly agree’. The item assessing additional time spent in completing mental health records reduced with time. While at T1, 62% of the health workers spent more than 10 min on reporting for additional mental health indicators, this was reduced to 28% at T2, when the response was selected as either agreed or strongly agreed. Agreement for the item on ‘not having enough time to complete additional questions for routine data collection’ also changed from 80% to 43% from T1 to T2, when ‘agree’ and ‘strongly agree’ responses were combined (see Table 4).

**Table 4:** Results from structured questionnaire

Survey questions	Responses	T1 N = 16 N (%)	T2 N = 9 N (%)
Currently involved in routine data collection?	Yes	9 (56.2)	7 (77.8)
	No	7 (44.8)	2 (22.2)
Received training on routine data collection?	Yes	16 (100)	9 (100)
	No	0	0
How many mental illness patients do you see on average in a week?	1 or fewer	3 (18.8)	3 (42.9)
	2–4 per week	8 (50.0)	3 (42.9)
	5–7 per week	3 (18.8)	0
	8–10 per week	1 (6.3)	0
	> 10 per week	1 (6.3)	1 (14.2)
How much time do you think you spend, on average, with each patient who has mental health problems?	< 5 min	0	1 (14.3)
	5–10 min	2 (12.5)	1 (14.3)
	11–20 min	0	1 (14.3)
	21–30 min	2 (12.5)	0
	> 30 min	12 (75.0)	4 (57.1)
How much time do you spend on average recording information for *any* patient?	< 5 min	1 (6.3)	0
	5–10 min	2 (12.5)	4 (57.1)
	11–20 min	7 (43.8)	1 (14.3)
	> 20 min	6 (37.5)	2 (28.6)
How much *additional* time was needed using the new format?	< 5 min	0	1 (14.3)
	5–10 min	6 (37.5)	4 (57.1)
	11–20 min	3 (18.8)	0
	> 20 min	7 (43.8)	2 (28.6)
How satisfied are you with the new format?	Very dissatisfied	0	0
	A little dissatisfied	1 (6.3)	2 (28.6)
	Neutral	3 (18.8)	0
	A little satisfied	8 (50.0)	2 (28.6)
	Very satisfied	4 (25.0)	3 (42.9)
It is possible to use the mental health indicators in routine practice	Strongly disagree	0	0
	Disagree	1 (6.3)	4(44.4)
	Neutral	0	0
	Agree	8 (50.0)	3 (33.3)
	Strongly agree	7 (43.8)	2 (22.2)
I do not have enough time to complete additional questions for routine data collection.	Strongly disagree	0	0
	Disagree	1 (6.3)	3 (42.9)
	Neutral	2 (12.5)	1 (14.3)
	Agree	10 (62.5)	3 (42.9)
	Strongly agree	3 (18.8)	0
Routine mental health data collection is important	Strongly disagree	0	0
	Disagree	0	1 (11.1)
	Neutral	0	1 (11.1)
	Agree	1 (6.3)	6(66.7)
	Strongly agree	15 (93.8)	1 (11.1)
I am interested and willing to be involved in the data collection using these formats	Strongly disagree	0	0
	Disagree	0	0
	Neutral	0	0
	Agree	4 (25.0)	8 (88.9)
	Strongly agree	12 (75.0)	1 (11.1)
The data collection format is easy to understand	Strongly disagree	0	0
	Disagree	0	0
	Neutral	0	1 (11.1)
	Agree	12 (75.1)	4 (44.4)
	Strongly agree	4 (25.0)	4 (44.4)
The individual items appear relevant and useful	Strongly disagree	0	0
	Disagree	0	0
	Neutral	0	0
	Agree	1 (6.3)	5 (83.3)
	Strongly agree	15 (93.8)	1 (16.7)
I believe this form should be part of our normal work	Strongly disagree	0	0
	Disagree	1 (6.3)	2 (25.0)
	Neutral	0	0
	Agree	8 (50.0)	6 (75.0)
	Strongly agree	7 (43.8)	0
I feel confident using the data collection format	Strongly disagree	1 (6.3)	0
	Disagree	0	2 (22.2)
	Neutral	8 (50.0)	0
	Agree	7 (43.8)	4 (44.4)
	Strongly agree	0	3 (33.3)

Accuracy amongst completed patient records was generally high (Table 3). For indicators on diagnosis, exact diagnosis and severity, the correction of completion rate was 96.7% at T1 and 95.2% at T2. The indicator on treatment however showed an increase of 28.4% in correction of completion column when measured from T1 to T2. Percentage of incorrectly completed records, attributed to the illogical correction of completion, also reduced across the two time points.

### Organisational *determinants*

#### Overstretched health workers

Nurses at the primary facilities managed the new mental health programme, which included responsibilities for counselling, reporting and overall management. During the interviews, programme managers confirmed that directives from the Department of Health to allocate two nurses per facility enabled the initiation of the documentation (using screening, case and follow up registers) in these facilities.

Most of the practitioners at primary care reported that nurses are competent enough to lead data collection tasks within mental health programmes. However, in view of staff shortages (in almost all facilities) and other priorities such as managing deliveries, medical officers and in charge medical officers reported it is difficult for nurses to complete registers and conduct counselling sessions for patients with mental disorders, affecting the feasibility and sustainability of these measures. A respondent stated:

*“Nurses have a role in every other National Health Programme and alone they handle many duties. There should be more nurses for this new programme. We try to relieve nurses for few hours of a day but in my understanding other health workers like AHSA (Accredited Social Health Activist) can also do the job of reporting or counselling in my facility.” * (Medical Officer, Ichhawar).

Interestingly, staff perception of the burden due to the new forms also increased over time. Within the responses during structured questionnaires, health workers reported a reduction in percentage of agreement from around 90% at T1 to 75% at T2 when asked about whether they see the new mental health forms being a part of their routine work (Table 4).

#### Poor service delivery at the health facilities as a barrier to reporting

All supervisors and coordinators reported that the state government’s plan to expand mental health services across all districts in Madhya Pradesh affected mental health service delivery in the seven mental health scale up sites. Health workers said that the poor service delivery in the initial months was also influenced by delays in procuring essential medicines, mental health registers and referral slips at scale-up facilities, which in turn delayed reporting in the new registers. A nurse pointed out:

*“Our block medical officer has assigned me to manage Mann Kaksh (Mental Health Cell) and I like it here. I want to do the reporting, but it is difficult to get time to screen patients in outpatient hours as there are so many other patients of fever and cold and then these patients [mental disorders] expect medicine. We have only 100 olanzapine till now. Sir [programme coordinator] has been assuring us we will soon get them. So, for me reporting is not a problem. But we need other things.” * (Nurse, Ashta).

Another health worker points out that alongside other procurement issues, unavailability of doctors also led to poor identification and diagnosis of people with Depression, Psychosis and Alcohol Use Disorders in her facility:

*“We don’t have much to report for these patients. Not all doctors send patients to Man Kaksha [Mental Health Cell]. Reporting we can do after our breaks also but sometimes there are no patients throughout the weeks.” * (Health worker, Nasrullaganj).

#### Facilitation of mental health reporting in facilities

Most nurses found the initial 3-day training session was easy to follow and user-friendly, as reported in the qualitative interviews. Monthly on-the-job supervision on how to populate a field in the new registers greatly assisted the implementation of indicators, as indicated by several respondents to the interviews.

Ensuring co-ordination between nurses, medical officers and the psychiatrist, maintaining referral linkages with tertiary care, continuous supportive supervision of the nurses and collaboration with the other health information system staff at facilities, were all suggested to have facilitated reporting. A nurse reported:

*“He [programme coordinator] visits us every month or sometimes twice in one month so we do ask him as and when we don’t understand any fields in the form, in the first month I had to rework on all the forms and he showed me how to do that.” * (Nurse 2, District Hospital).

Another nurse mentions how peer support has also helped them in records completion:

*“All 4 of us [nurses] took the induction together, we have taken many such trainings together. One of the nurses lives close to my house and as I was not there when coordinators came to teach at the CHC, so the other nurse has helped me complete registers. All these registers were made by us before we got the nicer ones from the government.” * (Nurse 1, Rehti).

#### Integration and adoption of mental health forms within general health information system

Practitioners at primary care elaborated on their experiences of integration of other similar programmes in the past and attributed the success to the project staff investing in enhancing staff motivation, ongoing engagement with the health facility and engaging staff in community awareness programmes. A medical officer stated:

*“You all might think it is tough to sustain a programme, but in my 15* *years’ experience in seeing people bring an agenda, do some implementation and go back. What has worked in my Community Health Centre is if they do not dictate but help and work it out with my staff, I have seen that in TB and other programmes, my staff here are all competent.” * (Medical officer 2, Nasrullaganj).

Supervisors and coordinators supported the concept of integration of mental health information with the routine information system. Further, they commented on the comorbid disease burden and the need for overall integration at primary care level for sustainable measures.

According to the programme coordinators, the simplicity of the new indicators can help in the integration. However, a few programme managers said different strategies will be required to achieve integration of these forms at state/district or national level. For example, one programme coordinator suggested that, for integration to occur below district level, training modules for health management information systems will be needed.

### Behavioural *factors*

#### Importance of mental health information systems at the facilities

All nurses interviewed considered having a routine mental health information system in their facility important for revising patients’ treatment plans. Regarding this importance, one of the health workers stated:

*“[..] we record the new and the returning patients in the mental health main register. We know for example we must do depression testing with PHQ-9 forms. Every time the patient comes, we must do PHQ*-*9 again. So, all this need to go in the register. So, it is necessary to record all this information somewhere like what medications needs to be decreased or stopped. He [medical officer] needs it from time to time. In my opinion, this register is very helpful.” * (Health worker, Budhni).

The health workers, the HMIS staff and the programme coordinators largely reinforced the need for the new reporting formats. Structured questionnaires also revealed a 100% score when the response was marked as either agreed or strongly agreed, on the items capturing the relevance and usefulness of collecting mental health data in the new formats, at both time points.

#### Knowledge and attitudes of health workers

When the respondents were asked about how the new forms and training on mental health information system have helped them in their daily work, most reported to have improved knowledge and awareness of mental disorders.

*“… after the training on mental health data. I detect and report on depression cases, and alcohol cases [cases with alcohol use disorders] I gained knowledge on how to maintain an OPD register and write about how and what talked in the session [counselling session].” * (Nurse, Budhni).

Some participants in the qualitative studies said that the training was able to break negative attitudes and misconceptions towards people with mental disorders:

*“[…] I had initial hesitancy or fear you can say, in managing the mental health programme, but the trainings have helped build positive attitude for myself and for patients I felt such cases can be cured and have better lives.” * (Health worker 1, Rehti).

Similar to the qualitative results, the quantitative findings also suggest greater willingness of health workers to be involved in completing the new forms, which was maintained at both T1 and T2.

## Discussion

Integration of care for people with mental disorders is aided through system-wide healthcare approaches [25], such as strengthening routine mental health information systems. The provision of mental health services is affected by lack of information, as information is imperative for planning decisions and management practices [10]. As a result, the WHO developed a comprehensive procedure to strengthen mental health information systems globally [6]. There is an increasing support for the evaluation of health management information systems targeting several implementation outcomes including timeliness, relevance and accuracy [26]. However, to the best of our knowledge, our study is the first to evaluate the implementation of key mental health indicators at primary care facilities in India.

Evaluation of the new system of collecting mental health data at primary care facilities through mental health indicators demonstrated overall good performance, indicated by higher correctness of the completed records. First, this study found high completion rates for some of the indicators at T1, with only a slight reduction in their completion at T2. These included indicators on exact diagnosis, treatment administered, follow-up and referrals. However, items capturing severity required additional work and were hard to capture. Nevertheless, correctness with which mental health proformas were completed, remained high and constant across the time points. Therefore, over time the quality with which all of these indicators were completed remained high.

Second, perceived importance, user-friendliness, and need for the new mental health indicators, were all contributors to acceptability [21], which was high across health workers and their supervisors using the forms.

Third, health workers’ perception of burden increased and there was a slight dip in their feeling of being positive about reporting. Therefore, even with the state level buy- in (for example by releasing state directives to relieve nurses for mental health programme activities), additional reporting as a result of newly introduced indicators and clinical responsibilities left health workers overburdened.

Fourth, continued supervision and support emerged as a key strategy which maintained positivity and ensured feasibility of the new mental health indicators and forms. Thus, a support system of coaching and providing technical assistance is a part of the design focused implementation, which included installing, optimising and improving mental health service delivery and support systems at primary care facilities within the PRIME programme [20]. External support and supervision for data collectors has also enabled different stages of developing mental health information systems in Ghana and South Africa [27]. Considering that in this study the external support provided by research projects such as PRIME and Emerald was of limited duration, further long-term commitment of support is needed from the government. This contributes to the understanding around sustainability of these measures.

Fifth, this study supports the integration of mental health indicators in routine monitoring at primary care facilities through implementation strategies, such as developing and implementing a combined HMIS module which includes training on mental health indicators. However, the primary care staff needed continuous support from an external technical support team, which in our case was provided by PRIME programme. Integration of mental health indicators into district health information systems in two South African provinces have shown that the integration of mental health information system into pre-existing information systems enables the overall process of integration [27].

### Study limitations

There are several limitations of this study which should be considered while interpreting these findings. For the case records review, there were no internal validation checks conducted, however the quality of the records was assessed independently by the co-researchers. The structured questionnaire conducted with health workers and health managers might have introduced social desirability bias. Some of these questions were further explored qualitatively to cover any critical point of view. This study focused only on the data collection aspect of the mental health information systems. The overall system of collection, transmission, analysis, dissemination and use should be assessed for a complete evaluation of information systems for mental health programs. Additionally, some interviews were carried out in Hindi, and therefore some crucial information might have been lost during the process of translation. Lastly, this study focused on the assessment of health service delivery indicators only, and health system indicators such as indicators on psychotropic medicines, trainings etc. could not be evaluated due to time limitations. Lastly, the case records review assessed the completion of the indicators on the data collection forms, however the clinical accuracy of this information could not be assessed.

### Implications of the findings

The results of this study have several implications. First, it is necessary to understand that information systems alongside other health systems building blocks needs to be integrated with primary health care programmes for effective integration. This is particularly important in the case of mental health in LMICs where the treatment gap is above 90% [3]. This study suggests adoption of a set of mental health indicators at primary care facilities under the DMHP in India. Second, it appears that with some external technical support it is feasible to collect data on diagnosis, severity, treatment, follow-up and referrals for three priority disorders. Third, within the context of Sehore District, it is essential that these measures have buy-in from the government and that they are maintained and incorporated in the state/district level programme implementation plans or linked with other health care programmes at primary facilities. Fourth, owing to overburdened health staff and limited support from research projects, the sustainability of such measures needs to be further researched in real time. Future research should evaluate the integrated information systems at primary care facilities, especially as the need for mental health surveillance is rising.

## Conclusions

This study evaluated the implementation of new indicators for monitoring mental health care in primary care facilities in India. It demonstrates that the implementation of seven mental health indicators by health workers at primary care facilities in Sehore District is feasible, with high levels of completion and correctness of completion, as well as high levels of perceived acceptability by health workers using the new indicators. Technical assistance from the project team was imperative in maintaining perceived utility of these indicators. By and large, this study supports system wide measures to strengthen (and integrate) mental health information systems, which are needed to monitor the progress of community mental health programmes and to scale up mental health interventions at primary care in LMICs. However, long-term support and buy-in from the governments is needed to sustain these measures.

## Data Availability

The datasets used and/or analysed during the current study are available from the corresponding author on reasonable request.

## Abbreviations

HMIS: Health Management Information Systems
LMIC: Lower- and Middle-Income Countries
PRIME: Programme for Improving Mental Health Care
Emerald: Emerging Mental Health Systems in Low- and Middle-Income Countries
PRISM: Performance of Routine Information System Management
DMHP: District Mental Health Programme
DoH: Department of Health
MoHFW: Ministry of Health and Family Welfare
